# Circular RNA hsa_circ_0000277 sequesters miR-4766-5p to upregulate LAMA1 and promote esophageal carcinoma progression

**DOI:** 10.1038/s41419-021-03911-5

**Published:** 2021-07-05

**Authors:** Peng Li Zhou, Zhengyang Wu, Wenguang Zhang, Miao Xu, Jianzhuang Ren, Qinhui Zhang, Zhanguo Sun, Xinwei Han

**Affiliations:** Department of Interventional Radiology, The First Affiliated Hospital of Zhengzhou University, Zhengzhou, Henan China

**Keywords:** Cancer genomics, Cell invasion, Prognostic markers

## Abstract

Growing evidence has indicated that circular RNAs (circRNAs) play a pivotal role as functional RNAs in diverse cancers. However, most circRNAs involved in esophageal squamous cell carcinoma (ESCC) remain undefined, and the underlying molecular mechanisms mediated by circRNAs are largely unclear. Here, we screened human circRNA expression profiles in ESCC tissues and found significantly increased expression of hsa_circ_0000277 (termed circPDE3B) in ESCC tissues and cell lines compared to the normal controls. Moreover, higher circPDE3B expression in patients with ESCC was correlated with advanced tumor-node-metastasis (TNM) stage and dismal prognosis. Functional experiments demonstrated that circPDE3B promoted the tumorigenesis and metastasis of ESCC cells in vitro and in vivo. Mechanistically, bioinformatics analysis, a dual-luciferase reporter assay, and anti-AGO2 RNA immunoprecipitation showed that circPDE3B could act as a competing endogenous RNA (ceRNA) by harboring miR-4766-5p to eliminate the inhibitory effect on the target gene laminin α1 (*LAMA1*). In addition, *LAMA1* was significantly upregulated in ESCC tissues and was positively associated with the aggressive oncogenic phenotype. More importantly, rescue experiments revealed that the oncogenic role of circPDE3B in ESCC is partly dependent on the miR-4766-5p/LAMA1 axis. Furthermore, bioinformatics analysis combined with validation experiments showed that epithelial-mesenchymal transition (EMT) activation was involved in the oncogenic functions of the circPDE3B–miR-4766-5p/LAMA1 axis in ESCC. Taken together, we demonstrate for the first time that the circPDE3B/miR-4766-5p/LAMA1 axis functions as an oncogenic factor in promoting ESCC cell proliferation, migration, and invasion by inducing EMT, implying its potential prognostic and therapeutic significance in ESCC.

## Background

Esophageal squamous cell carcinoma (ESCC) is ranked eighth among the most common aggressive neoplasms and sixth among the principal causes of cancer-related death around the world [1]. In 2012, there were about 455,800 new ESCC cases and 400,200 deaths globally [2]. AS there are few precise biomarkers, most patients with ESCC are diagnosed only when it progresses to the intermediate or advanced stage. Consequently, despite the progressive advancements in multimodality therapies, ESCC prognosis remains poor [3]. Furthermore, ESCC molecular targeted therapies are far from satisfactory, mainly because its molecular pathogenesis is currently generally uncertain. Consequently, there is an urgent need to identify applicable therapeutic targets and molecular biomarkers for early-stage diagnosis to improve the clinical outcomes in patients with ESCC.

Circular RNAs (circRNAs) are a new class of endogenous noncoding RNAs (ncRNAs) characterized by a continuous covalently closed loop without 5′ or 3′ end structure [4]. CircRNAs are widely expressed in diverse human tissues, and their expression is highly stable in comparison to the expression of their linear RNA isoforms [5]. Increasing evidence indicates that circRNAs may have important roles in many physiological and pathophysiological processes and act as protein decoys, microRNA (miRNA) sponges, and as regulators of transcription and splicing [6]. Recently, numerous reports revealed that circRNAs are frequently deregulated and play important roles in tumor initiation and development in distinct human cancers, including ESCC [7]. For example, Chen et al. [8] found that silencing circNTRK2 suppressed ESCC proliferation and invasion. Li et al. [9] reported that ciRS-7 accelerated ESCC growth and metastasis through the miR-7/HOXB13 axis. However, our understanding of the specific roles of circRNAs in ESCC remains limited, and further exploration is warranted.

Epithelial–mesenchymal transition (EMT) is a highly conserved cellular process characterized by the loss of intercellular junctions and epithelial characteristics, and the demonstration of an enhanced mesenchymal phenotype [10]. Mounting evidence shows that EMT contributes to cancer progression and metastasis [11]. Furthermore, a growing body of evidence indicates that multiple circRNAs, such as circEPSTI1 [12] and circRNA_0000140 [13] (oral squamous cell carcinoma), circREPS2 [14] (gastric cancer), circPTCH1 [15] (renal cell carcinoma), circ_0003998 [16] (hepatocellular carcinoma), and circHMCU [17] (breast cancer), participate in cancer metastasis by influencing EMT. These findings suggest that certain circRNAs play important roles in promoting cancer metastasis through the regulated EMT process. Therefore, it is believed that circRNAs can provide new insight into the molecular mechanisms of cancer metastatic progression, and might also identify new treatment targets for patients with metastatic-stage ESCC.

## Results

### circPDE3B expression was upregulated in ESCC tissues and cell lines

**Fig. 1:**
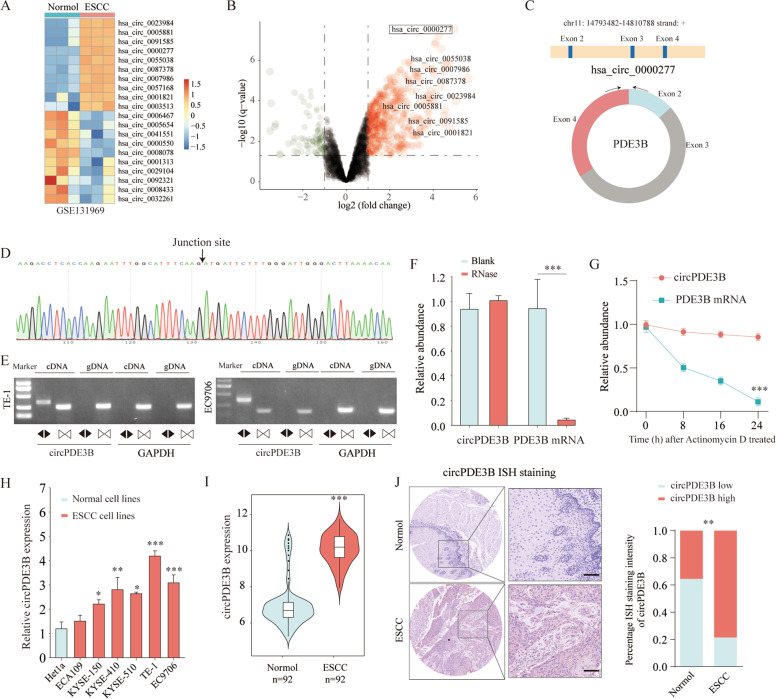
Identification of differentially circular RNAs in ESCC. **A** Clustered heat map of the differentially expressed circRNAs in three pairs of human ESCC cancerous tissues and adjacent normal tissues (GSE131969). circRNAs with increased (yellow) or decreased (blue) expression are presented. **B** Volcano plots comparing the expression of circRNAs in ESCC cancerous tissues with adjacent normal tissues. The color indicated dots represent the circRNAs upregulated (red dots) and downregulated (green dots) by 1.5-fold and *p* values < 0.05. **C** The genomic loci of the PDE3B gene and circPDE3B. **D** The back-splice junction site of circPDE3B was validated by Sanger sequencing. **E** RT-PCR assay with divergent or convergent primers indicated the existence of circPDE3B in ESCC tissue. GAPDH was used as a negative control. **F** qRT-PCR analysis of PDE3B mRNA and circPDE3B after treatment with RNase R in EC9706 cells. **G** qRT-PCR analysis of PDE3B mRNA and circPDE3B in EC9706 cells after treatment with Actinomycin D. **H** Expression levels of circPDE3B in 92 paired ESCC specimens and the corresponding paired normal adjacent tissues. **I** Expression levels of circPDE3B determined by qRT-PCR in mammary normal cell lines and ESCC cell lines. **J** Representative images and analysis results of circPDE3B expression in ESCC tissues were detected by ISH assays. Scale bar, 200 μm. *p < 0.05, **p < 0.01, ***p < 0.001.

We analyzed three paired ESCC tissue samples to investigate the ESCC tissue circRNA expression profile using a circRNA microarray from Gene Expression Omnibus (GSE131969). There was significantly increased expression of hsa_circ_0000277, also termed circPDE3B, in ESCC tissues as compared with the matched nonneoplastic counterparts (Fig. 1A, B; Supplementary Table S2). Comparison of the *PDE3B* mRNA sequences with the expected circPDE3B sequences from circBase showed that circPDE3B was looped and comprised exons 2–4 of its parental gene (Fig. 1C). We used Sanger sequencing to confirm the head-to-tail splicing (Fig. 1D). We also designed convergent and divergent primers for amplifying the linear and circRNA based on TE-1 and EC9706 cell complementary DNA (cDNA) and genomic DNA (gDNA) using real-time polymerase chain reaction (RT-PCR). circPDE3B could only be amplified in cDNA, but not in gDNA (Fig. 1E). We used RNase R to confirm circPDE3B stability. The RNase R sharply decreased the levels of the linear forms of *PDE3B*, but did not digest circPDE3B (Fig. 1F). Moreover, resistance to actinomycin D confirmed the circular structure of circPDE3B (Fig. 1G).

Next, we investigated circPDE3B expression in ESCC cell lines by quantitative RT-PCR (qRT-PCR). circPDE3B expression was upregulated in ESCC cell lines normalized to esophageal epithelial cells (Fig. 1H). Moreover, qRT-PCR detected higher circPDE3B expression in 92 paired ESCC samples relative to the adjacent normal samples (Fig. 1I). Elevated circPDE3B expression was confirmed using in situ hybridization (ISH) on a tissue microarray (TMA) (Fig. 1J). Therefore, our subsequent experiments focused on the role of circPDE3B in ESCC progression.

### Upregulated circPDE3B expression was correlated with poor clinical outcomes of ESCC

**Fig. 2:**
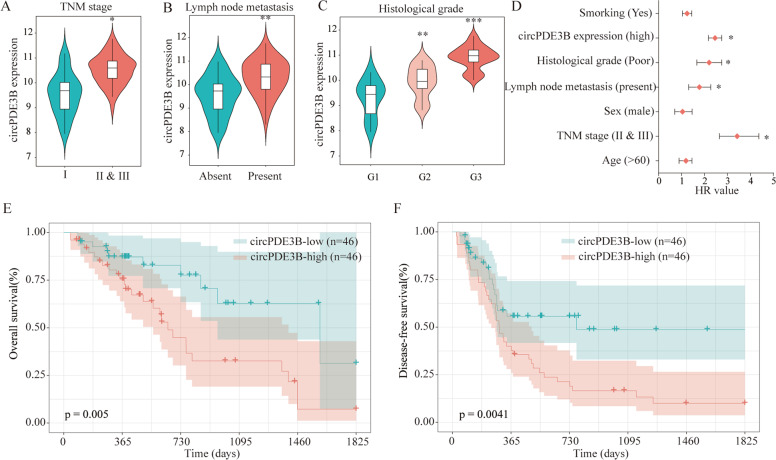
Upregulated circPDE3B was positively correlated with the poor prognosis of ESCC. The correlation between circPDE3B expression lever and TNM stage (**A**), lymph node metastasis (**B**), and histological grade (**C**) of ESCC patients. **D** The univariate Cox proportional hazards analysis was performed to depict the correlations between the indicated clinical criteria and disease-free survival rate. Patients with higher circPDE3B expression had a shorter overall survival time (**E**) and disease-free survival time (**F**) than those with lower circ-circPDE3B expression. *p < 0.05, **p < 0.01, ***p < 0.001.

To examine the correlation between circPDE3B expression and ESCC clinicopathological characteristics, patients with ESCC (*n* = 92) were classified into low- and high-circPDE3B expression groups. The clinicopathological factors between the two groups were assessed using the chi-square test. Fig. 2A–C show that higher circPDE3B expression correlated positively with lymphatic metastasis, advanced tumor-node-metastasis (TNM) stage, and poor histological grade. Univariate Cox proportional hazards analysis of the disease-free survival (DFS) rate showed that circPDE3B expression was an important prognostic factor (Fig. 2D); Multivariate analysis showed that circPDE3B were independent prognostic indicators for DFS (Supplementary Table S3). Furthermore, Kaplan–Meier analysis showed that patients with ESCC with high circPDE3B expression exhibited poorer OS and DFS rates compared with patients with low circPDE3B expression (Fig. 2E, F). These results indicate that the upregulated circPDE3B participates in ESCC progression and could be a potential prognostic target.

### circPDE3B promoted ESCC cell growth and invasion in vitro

**Fig. 3:**
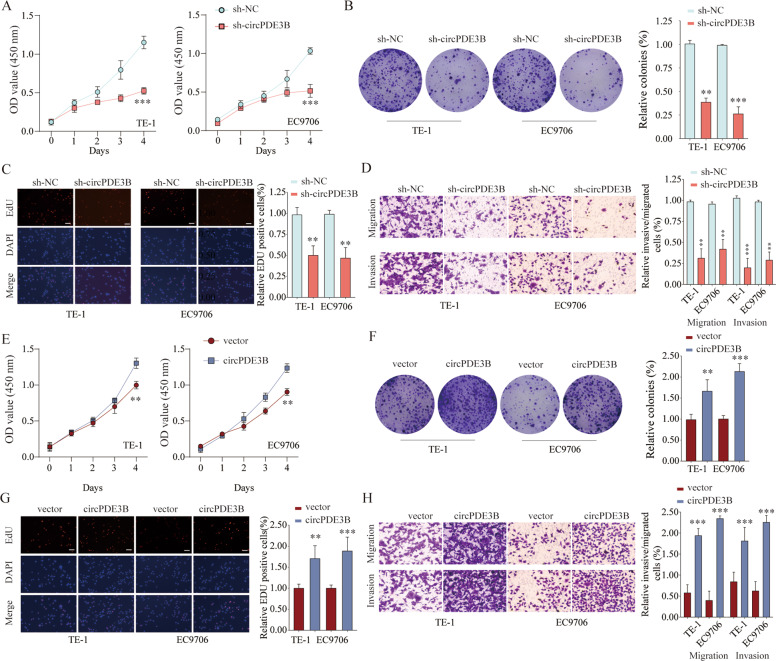
circPDE3B promotes ESCC cell proliferation and invasion in vitro. **A** CCK-8 assay was used to detect cell viability of TE-1 and EC9706 cells after circPDE3B knocking down. **B** Colony formation assays were used to detect the clone ability of TE-1 and EC9706 cells after circPDE3B knocking down. **C** EdU assays were used to detect the DNA synthesis rate of TE-1 and EC9706 cells after circPDE3B knocking down. Scale bar: 50 μm. **D** Transwell assays were used to detect cell migration and invasion capacities of TE-1 and EC9706 cells after circPDE3B knocking down. **E** CCK-8 assays were used to detect cell viability of TE-1 and EC9706 cells after enforced expression of circPDE3B. **F** Colony formation assays were used to detect the clone ability of TE-1 and EC9706 cells after enforced expression of circPDE3B. **G** EdU assays were used to detect the DNA synthesis rate of TE-1 and EC9706 cells after enforced expression of circPDE3B. Scale bar: 50 μm. **H** Transwell assays were used to detect cell migration and invasion capacities of TE-1 and EC9706 cells after enforced expression of circPDE3B. *p < 0.05, **p < 0.01, ***p < 0.001.

We used the loss- and gain-of-function strategy to investigate the biological function of circPDE3B in ESCC cells. First, we designed three sh-circPDE3B products specifically targeting the circPDE3B junction sites, and transfected them into TE-1 and EC9706 cells. The short hairpin RNAs (shRNAs) significantly silenced circPDE3B expression, whereas *PDE3B* mRNA expression was unaltered (Supplementary Fig. S1). Among the shRNAs, knockdown efficiency was best by sh-circPDE3B#03. Next, we evaluated cell viability using the Cell Counting Kit-8 (CCK-8) assay and found that circPDE3B knockdown suppressed TE-1 and EC9706 cell proliferative ability significantly (Fig. 3A). In addition, the colony formation assay demonstrated that circPDE3B had an important influence on preserving high colony-forming ability (Fig. 3B). Consistently, the EdU (ethynyl deoxyuridine) assay showed that circPDE3B knockdown inhibited the DNA synthesis rate of ESCC cells (Fig. 3C). Moreover, Transwell assays demonstrated significantly fewer migrated and invaded cells in the sh-circPDE3B group compared with the sh-NC (negative control) group (Fig. 3D). In addition, metastasis-associated proteins MMP-2, MMP-7, and MMP-9 were decreased in circPDE3B knockdown cells (Supplementary Fig. S2). Conversely, circPDE3B overexpression correlated with a notable increase in cell growth abilities (Fig. 3E–G) and greatly enhanced migration and invasive potential in TE-10 and KYSE-410 cells (Fig. 3H). Collectively, these data suggest that circPDE3B can promote cell proliferation and significantly enhance ESCC cell invasion and migration in vitro.

### circPDE3B enhanced ESCC cell growth and invasion in vivo

**Fig. 4:**
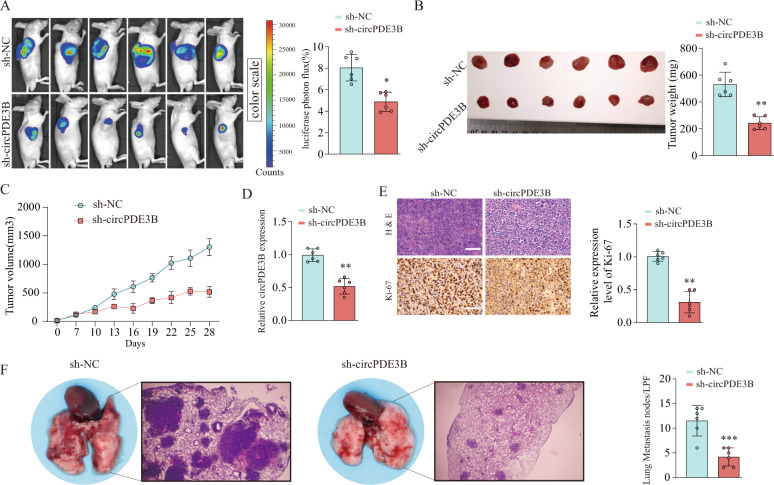
circPDE3B promotes ESCC cell growth and metastasis in vivo. **A** Representative bioluminescent images and quantification of bioluminescent imaging signal intensities in nude mice from sh-NC or sh-circPDE3B group. Comparison of tumor weight (**B**) and volume (**C**) in nude mice subcutaneously implanted with sh-NC/EC9706 or sh-circPDE3B/EC9706 cells (*n* = 6/group). **D** circPDE3B expression levels in xenograft tumors were determined by qRT-PCR. **E** Immunohistochemical assay of Ki-67 in subcutaneously formed tumors in the sh-NC or sh-circPDE3B groups. Scale bar: 200 μm. **E** Pulmonary metastases of mice intravenously injected with sh-NC/EC9706 or sh-circPDE3B/EC9706 cells. Lung micrometastatic nodes in per low power field (LPF) were counted. *p < 0.05, **p < 0.01, ***p < 0.001.

Next, we validated the inhibitory effect of silencing circPDE3B on tumor growth and metastasis by subcutaneously injecting circPDE3B knockdown EC9706 cells into the right flanks of nude mice to establish a xenograft tumor model, and injecting the mouse lateral tail vein to establish a model of lung metastasis. We used an in vivo imaging system to monitor the tumor growth dynamically every 3 days. circPDE3B knockdown resulted in significantly decreased fluorescence signals in the subcutaneous tumors (Fig. 4A). In addition, the tumors in the mice inoculated with circPDE3B-silenced EC9706 cells developed more slowly than that in the mice inoculated with control cells, with markedly low tumor weight and smaller tumor volume (Fig. 4B, C). qRT-PCR detected lower circPDE3B expression in circPDE3B-silenced xenograft tumors (Fig. 4D). Moreover, the xenograft tumors underwent immunohistochemical (IHC) staining of Ki-67, which is used as a proliferation marker. circPDE3B-silenced xenograft tumors had fewer Ki-67-positive nuclei than the negative control (Fig. 4E). Furthermore, the lungs of the mice inoculated with circPDE3B-silenced EC9706 cells had fewer and smaller micrometastatic lesions than those inoculated with NC cells (Fig. 4F). Collectively, these results indicate that circPDE3B knockdown can inhibit ESCC cell growth and metastatic potential in vivo.

### circPDE3B exerted its function by sponging miR-4766-5p

**Fig. 5:**
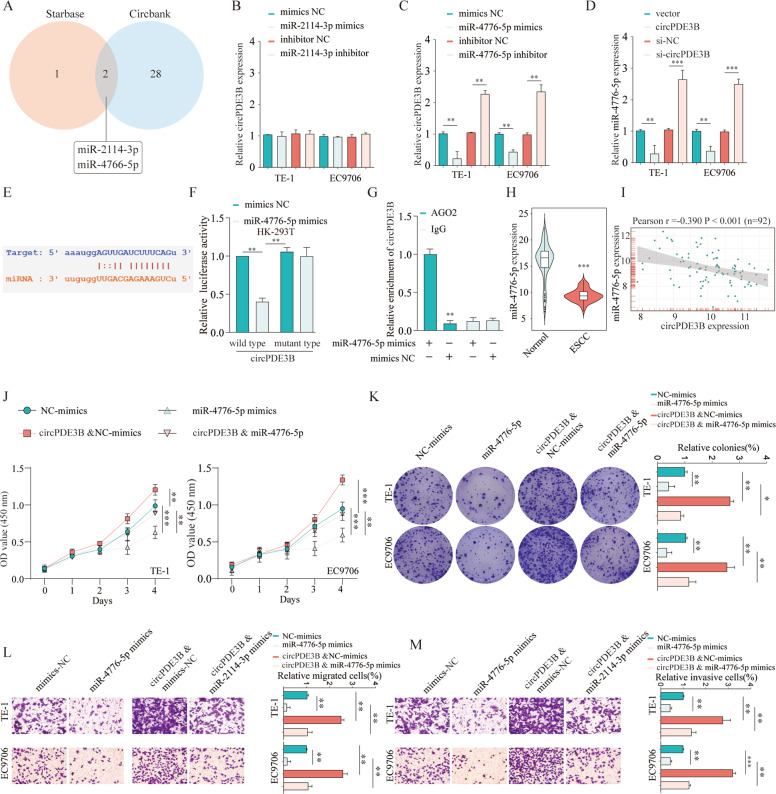
circPDE3B exerts its oncogenic function by sponging miR-4766-5p. **A** The intersection of miRNAs targeting circPDE3B predicted by Circbank and Starbase is shown via the Venn diagram. **B**, **C** The relative expression of circPDE3B in TE-1 or EC9706 cells after transfected miR-2114-3p or miR-4766-5p was analyzed by qRT-PCR, respectively. **D** The relative expression of miR-4766-5p in TE-1 or EC9706 cells after circPDE3B knocking down or overexpression was analyzed by qRT-PCR, respectively. **E** Schematic of the predicted binding site between miR-4766-5p and circPDE3B sequence. **F** Cells were transfected with a luciferase reporter plasmid containing wide-type circPDE3B or mutant-type circPDE3B, together with or without miR-4766-5p. The relative luciferase activity was analyzed. **G** Immunoprecipitation of AGO2 (control, anti-IgG) in miR-4766-5p mimics or mimics control transfected ES9706 cells were performed. **H** Expression levels of miR-4766-5p in 92 paired ESCC specimens and the corresponding paired normal adjacent tissues. **I** Correlation analysis of circPDE3B and miR-4766-5p in ESCC patients’ tissues. **J** CCK-8 assays were used to detect cell viability of TE-1 and EC9706 cells after indicated transfection. **K** Colony formation assays were used to detect the clone ability of TE-1 and EC9706 cells after indicated transfection. Transwell assays were used to detect cell migration (**L**) and invasion (**M**) capacities of TE-1 and EC9706 cells after indicated transfection. *p < 0.05, **p < 0.01, ***p < 0.001.

circRNA acts as a miRNA sponge to regulate miRNA targets [6]. RNA fluorescence ISH (FISH) showed predominately cytoplasmic localization of circPDE3B (Supplementary Fig. S3). Therefore, we further address whether circPDE3B sponges miRNAs in ESCC cells. We select two candidate miRNAs by overlapping the prediction results of the circPDE3B sequence miRNA recognition elements through starBase and circBank (Fig. 5A). Then, we examined whether the candidate miRNAs could bind circPDE3B directly. qRT-PCR indicated that miR-4766-5p, but not miR-2114-3p, significantly downregulated circPDE3B expression in the ESCC cells (Fig. 5B, C). In addition, circPDE3B overexpression decreased miR-4766-5p expression, while circPDE3B knockdown enhanced it (Fig. 5D). We also predicted the miR-4766-5p and circPDE3B binding site (Fig. 5E) and used a dual-luciferase reporter assay to detect circPDE3B luciferase intensity. The luciferase intensity of wild-type circPDE3B was significantly inhibited by miR-4766-5p, but the mutant circPDE3B was unaffected (Fig. 5F). Furthermore, the AGO2 immunoprecipitation assays indicated that, compared with the NC group, the miR-4766-5p-captured fraction had >5-fold enrichment of circPDE3B (Fig. 5G). Moreover, the ESCC tissues had significantly decreased miR-4766-5p expression compared with the normal tissues (Fig. 5H). miR-4766-5p and circPDE3B expression levels in ESCC tissues were correlated negatively (Fig. 5I). Taken together, circPDE3B specifically sponges miR-4766-5p.

After validating the interaction between circPDE3B and miR-4766-5p, we detected whether the oncogenic functions by circPDE3B are attributed to its repression effect on miR-4766-5p. the proliferative abilities of the transfected TE-1 and EC9706 cells were significantly inhibited by miR-4766-5p overexpression, while co-transfection with the circPDE3B vector partially reversed the inhibitory effects (Fig. 5J, K). Similarly, circPDE3B upregulation reversed the decreased number of migrated and invasive cells induced by ectopic miR-4766-5p expression (Fig. 5L, M). These results show that circPDE3B promotes the aggressive oncogenic phenotype of ESCC cells, at least in part, by modulating miR-4766-5p.

### circPDE3B regulated *LAMA1* by sequestering miR-4766-5p

**Fig. 6:**
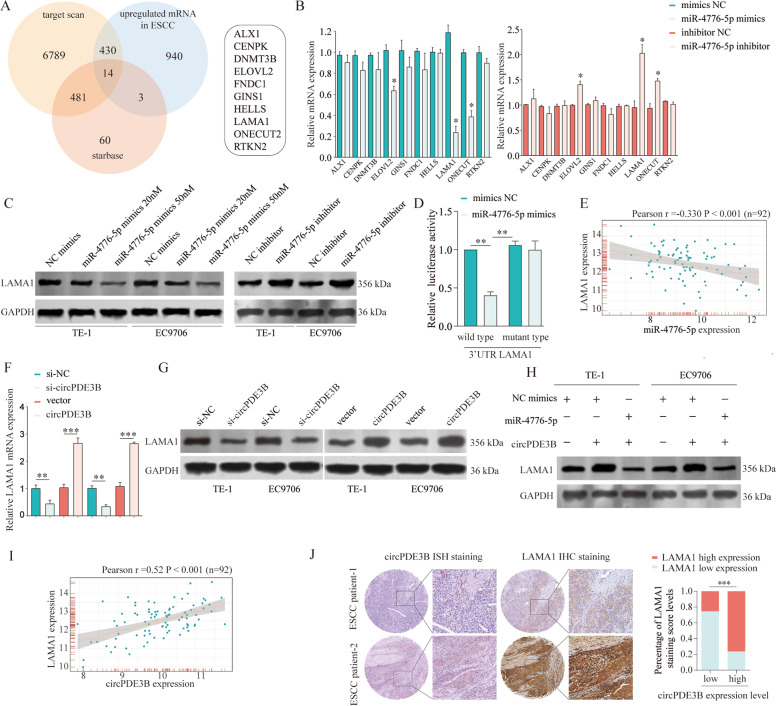
circPDE3B indirectly enhances LAMA1 expression through sponging miR-4766-5p. **A** The intersection of mRNAs potential targeting by miR-4766-5p predicted by Targetscan, Starbase, and mRNAs upregulated in TCGA ESCC database is shown via Venn diagram. **B** The relative expression of candidate mRNAs in EC9706 cells was analyzed by qRT-PCR after transfected miR-4766-5p mimics or inhibitors, respectively. **C** The relative protein expression of LAMA1 in TE-1 or EC9706 cells was analyzed by western blot after transfected miR-4766-5p mimics or inhibitor, respectively. **D** Cells were transfected with a luciferase reporter plasmid containing wide-type or mutant-type 3′UTR sequence of LAMA1, together with or without miR-4766-5p. The relative luciferase activity was analyzed. **E** Correlation analysis of LAMA1 and miR-4766-5p in ESCC patients’ tissues. **F**, **G** The expression of LAMA1 in ESCC cells was analyzed by qRT-PCR or western blot after transfected circPDE3B siRNA or vector, respectively. **H** The expression of LAMA1 in ESCC cells was analyzed by western blot after circPDE3B overexpression with or without miR-4766-5p mimics transfection. **I** Correlation analysis of LAMA1 and circPDE3B in ESCC patients’ tissues. **J** Co-expression of LAMA1 and circPDE3B in ESCC tissues were determined by IHC or ISH staining, respectively. *p < 0.05, **p < 0.01, ***p < 0.001.

circPDE3B could act as a miRNA sponge, thereby preventing miRNAs from binding and negatively regulating their target genes. Subsequently, we used two algorithms (TargetScan and starBase) to identify the putative targets genes of miR-4766-5p, while the candidate mRNAs upregulated in ESCC tissues were selected based on The Cancer Genome Atlas (TCGA) analysis. Ten candidate mRNAs (*ALX1*, *CENPK*, *DNMT3B*, *ELOVL2*, *FNDC1*, *GINS1*, *HELLS*, *LAMA1*, *ONECUT2*, and *RTKN2*) were selected for validation via qRT-PCR (Fig. 6A). miR-4766-5p regulated the mRNA levels of laminin α1 (*LAMA1*), *ELOVL2*, and *ONECUT2* (Fig. 6B), but a notable change was observed only for LAMA1 protein levels compared with the NC (Fig. 6C; Supplementary Fig. S4). We performed dual-luciferase reporter assays to confirm whether *LAMA1* and miR-4766-5p interacted directly. The transfected miR-4766-5p and wild-type *LAMA1* had significantly reduced luciferase reporter activity, but the mutant-type *LAMA1* did not have notably altered luciferase reporter activity (Fig. 6D). In addition, miR-4766-5p and *LAMA1* in ESCC tissues were negatively correlated by relativity analysis, suggesting possible binding ability (Fig. 6E).

Based on the above findings, we hypothesized that circPDE3B regulates *LAMA1* expression by sponging miR-4766-5p. qRT-PCR and western blotting indicated that circPDE3B knockdown significantly decreased *LAMA1* expression, whereas forcibly expressed circPDE3B markedly enhanced *LAMA1* expression in ESCC cells (Fig. 6F, G). Rescue experiments revealed that miR-4766-5p mimics partly reversed the promoter effect of circPDE3B overexpression on *LAMA1* expression (Fig. 6H). Furthermore, a correlation study of circPDE3B and LAMA1 expression in ESCC tissues revealed a significantly positive correlation (Fig. 6I). Meanwhile, stronger IHC staining intensity of LAMA1 was positively associated with elevated circPDE3B expression in ESCC tissues (Fig. 6J). Altogether, our findings verify our postulation that circPDE3B sponges miR-4766-5p, which subsequently increases *LAMA1* expression.

### *LAMA1* was upregulated and correlated with a poor prognosis of ESCC

**Fig. 7:**
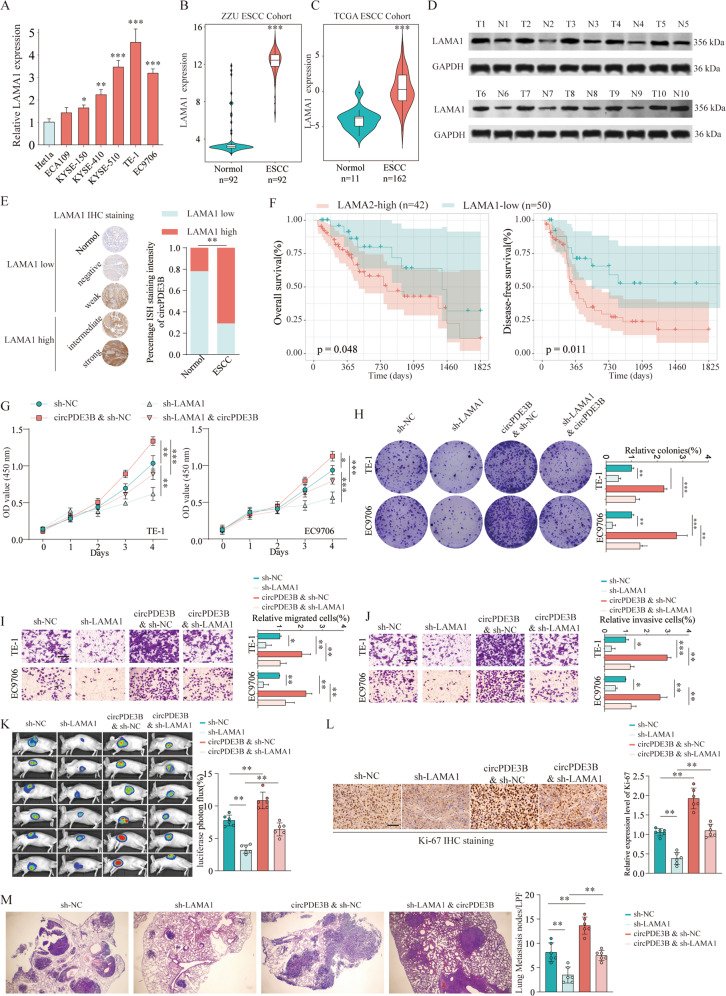
The oncogenic role of circPDE3B is partly dependent on the LAMA1. **A** Expression levels of LAMA1 determined by qRT-PCR in mammary normal cell lines and ESCC cell lines. **B** Transcriptional level of LAMA1 in ESCC tissues or non-tumor control tissues were analyzed by qRT-PCR in 92 paired ESCC specimens. **C** LAMA1 mRNA expression levels were analyzed in ESCC tissues from TCGA databases. **D** Protein levels of LAMA1 in ten paired ESCC and matched adjacent non-tumor tissues were determined by western blot. **E** Distribution of differently LAMA1 IHC staining intensive in ESCC tissue or non-tumor control tissues. **F** Kaplan–Meier plots of overall survival (OS) and disease-free survival (DFS) of patients with ESCC stratified by LAMA1 expression. CCK-8 assays (**G**) and colony formation assays (**H**) were used to detect cell viability of TE-1 and EC9706 cells after LAMA1 silencing with or without circPDE3B overexpression. Transwell assays were used to detect cell migration (**I**) and invasion (**J**) capacities of TE-1 and EC9706 cells after LAMA1 silencing with or without circPDE3B overexpression. **K** Representative bioluminescent images and quantification of bioluminescent imaging signal intensities in nude mice from indicated groups. **L** Immunohistochemical assay of Ki-67 in subcutaneously formed tumors from indicated groups. Scale bar: 200 μm. **M** Pulmonary metastases of mice intravenously injected with EC9706 cells after indicated transfection. Lung micrometastatic nodes in per low power field (LPF) were counted. *p < 0.05, **p < 0.01, ***p < 0.001.

To investigate the role of *LAMA1* in ESCC aggressive phenotypes, we performed qRT-PCR and the results indicated that *LAMA1* was overexpressed in the ESCC cell lines (Fig. 7A) and tissues (Fig. 7B). TCGA ESCC dataset yielded similar results (Fig. 7C). Similarly, western blotting of ten pairs of matched ESCC tissues and adjacent non-cancerous tissues showed significantly upregulated LAMA1 expression in the ESCC tissues (Fig. 7D). In addition, investigating LAMA1 expression in the tissues using immunohistochemistry showed that the ESCC tissues had stronger LAMA1 staining than the adjoining non-cancerous tissues (Fig. 7E). Moreover, patients with high LAMA1 expression had reduced mean days of OS and DFS compared with patients with low LAMA1 expression (Kaplan–Meier survival analysis, Fig. 7F). These findings all suggest that LAMA1 may play an oncogenic role in ESCC progression.

### The oncogenic role of circPDE3B was partly dependent on LAMA1

We verified whether the oncogenic activity of circPDE3B in ESCC was reliant on LAMA1. The CCK-8 and colony formation assays indicated that inhibiting LAMA1 expression impaired cell proliferation. However, co-transfection of the circPDE3B vector significantly reversed this inhibitory effect (Fig. 7G, H). Similarly, the compromised cell migration and invasive capabilities induced by LAMA1 silencing were abrogated following co-transfection with circPDE3B vector (Fig. 7I, J). In addition, to explore the effects of the circPDE3B/miR-4766-5p/LAMA1 axis on ESCC growth and metastasis in vivo, we established the following four EC9706 cell groups: NC (sh-NC), stable LAMA1 knockdown (sh-LAMA1), stable circPDE3B overexpression (circPDE3B and sh-NC), stable circPDE3B overexpression with LAMA1 inhibition (circPDE3B and sh-LAMA1). The in vivo tumor formation assay suggested that LAMA1 knockdown conspicuously inhibited tumor growth, while circPDE3B overexpression greatly promoted it when compared to the NC (Fig. 7K). In addition, suppression of LAMA1 partly abolished the promotional effect on tumor growth caused by circPDE3B overexpression. Moreover, LAMA1 silencing predominantly reduced Ki-67 expression, which could be reversed by circPDE3B reintroduction (Fig. 7L). Interestingly, LAMA1 downregulation greatly decreased metastatic tumor number and size, and the enforced circPDE3B expression again counteracted this change (Fig. 7M). Overall, our findings show that the oncogenic role of circPDE3B is partly dependent on LAMA1.

### The circPDE3B/miR-4766-5p/LAMA1 axis promoted ESCC progression by activating EMT

**Fig. 8:**
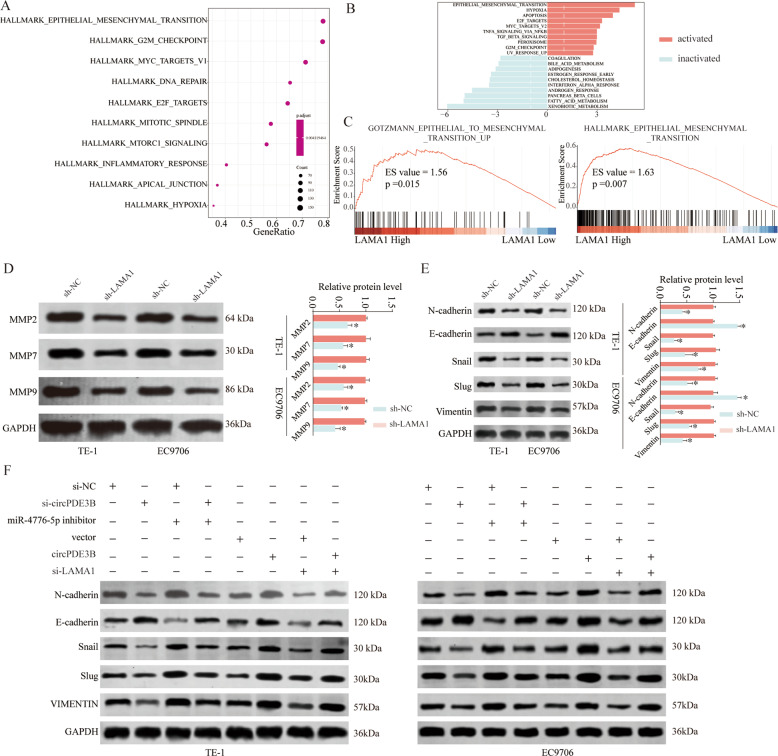
circPDE3B/miR-4766-5p/LAMA1 axis activates EMT. **A**, **B** KEGG pathway enrichment analysis of LAMA1 related pathways in TCGA ESCC dataset. **C** Gene set variation analysis (GSVA) and **D** the Gene Set Enrichment Analysis (GSEA) of the relationship between the expression level of LAMA1 and EMT-related gene signatures in the TCGA ESCC dataset. **D**, **E** Expression change of metastasis-related proteins (MMP-2/7/9) and EMT-related proteins (N-cadherin, E-cadherin, Snail, and Slug) and in ESCC cells after LAMA1 silencing or negative control. **F** Expression change of EMT-related proteins (N-cadherin, E-cadherin, β-catenin, Snail, Slug, and Vimentin) in ESCC cells after indicated transfection. *p < 0.05, **p < 0.01, ***p < 0.001.

We investigated the fundamental mechanism through which the circPDE3B/miR-4766-5p/LAMA1 axis contributes to ESCC progression. KEGG (Kyoto Encyclopedia of Genes and Genomes) analysis (Fig. 8A), gene set variation analysis (GSVA) (Fig. 8B), gene set enrichment analysis (GSEA) (Fig. 8C) indicated that high LAMA1 expression was closely and positively related to EMT activation, indicating that EMT may account for the oncogenic role of LAMA1 in ESCC. As expected, western blotting demonstrated decreased protein levels of MMP2/7/9, N-cadherin, Snail, Slug, and vimentin (mesenchymal molecule markers), and increased protein levels of E-cadherin (an epithelial molecule marker) after *LAMA1* knockdown (Fig. 8D, E). Moreover, similar inhibitory effects were observed after circPDE3B silencing (Fig. 8F). Rescue experiments revealed that miR-4766-5p inhibitor co-transfection significantly reversed the inhibitory effect on EMT induced by circPDE3B silencing (Fig. 8F), while LAMA1 siRNA co-transfection significantly reversed the promotional effect on EMT induced by circPDE3B overexpression. Consistently, IHC staining of xenograft tumor tissues yielded similar results (Supplementary Fig. S5). Collectively, our results reveal that the circPDE3B/miR-4766-5p/LAMA1 axis promotes ESCC progression by activating EMT.

## Discussion

ESCC is a major cause of cancer-related mortality around the world [1]. To date, treatment strategies for advanced ESCC are not effective, resulting in a poor 5-year survival rate. Although there has been encouraging progress in clarifying ESCC molecular mechanisms, patients with advanced ESCC nevertheless have unfavorable prognoses [3]. Therefore, revealing the underlying mechanism of ESCC is of great importance. The abnormal regulation of circRNAs is involved in the pathogenesis of human diseases and cancers [6]. A group of novel ncRNAs, circRNAs have a covalently closed-loop and act as essential regulators in human cancer, including ESCC [18, 19]. For example, CiRS-7 has a promoter role in ESCC growth and metastasis [9]. circNTRK2 levels were notably raised in ESCC and facilitated cell proliferation and invasion [8]. Wang et al. [20] found that circRNA-0008717 promoted ESCC progression by regulating the miR-203–Slug axis. Shi et al. [21] demonstrated that the circRNA LPAR3 acted as an oncogene in ESCC, enhancing ESCC cell malignancy by sponging miRNA-198. These reports all show that circRNAs have crucial roles in the progression of ESCC. Nevertheless, circRNA functions in ESCC remain largely unknown, and there are no reports on circPDE3B’s roles (hsa_circ_0000277) in cancer.

Here, we used circRNA microarray analysis to identify a novel circRNA, termed circPDE3B, which was significantly upregulated in human ESCC and correlated with clinical prognosis. Functionally, downregulating circPDE3B inhibited cell invasion and proliferation in vitro and in vivo, whereas forcibly expressed circPDE3B had an inductive role in ESCC. Therefore, our data indicate that circPDE3B might have a key role in ESCC pathogenesis and development. circRNA regulation of gene expression varies with the type of cancer or stages [6]. It is worth noting that circRNAs can function by acting as miRNA sponges to form a circRNA/miRNA/mRNA axis [6]. For example, Liu et al. reported that, in hepatocellular carcinoma, miR-328-5p was sponged by circRNA-5692, activating *DAB2IP* [22]. CircRNA_0000140 sponges miR-31 to suppress oral squamous cell carcinoma growth and metastasis [13]. CircRNA-002178 sponges miR-34 to enhance PDL1/PD1 expression to promote lung adenocarcinoma progression [23]. Bioinformatics analysis, a dual-luciferase reporter assay, and anti-AGO2 RNA immunoprecipitation proved the direct combination between circPDE3B and miR-4766-5p. We also found that miR-4766-5p was significantly downregulated in ESCC tissues. Rescue experiments suggested that the oncogenic activity of circPDE3B was dependent on the modulation of miR-4766-5p. In gastric cancer, miR-4766-5p is downregulated and significantly inhibits cell migration and invasion [24], which is consistent with our findings miR-4766-5p knockdown could enhance cell growth, metastasis, and chemoresistance remarkably [25]. Moreover, miR-4766-5p is also downregulated in colorectal cancer and may act as a tumor suppressor [26]. These findings show that circPDE3B acts as an oncogene in ESCC by sponging miR-4766-5p.

The ceRNA hypothesis states that circRNA can act as a ceRNA to regulate miRNA target gene expression. Bioinformatics analysis and validation experiments indicated that *LAMA1*, a subunit of laminin-1, is a potential miR-4766-5p target. circPDE3B overexpression increased both LAMA1 mRNA and protein, while miR-4766-5p mimics partially abolished these promoter effects. We also found significantly upregulated LAMA1 in ESCC tissues and that elevated LAMA1 correlated with shorter OS. Furthermore, functional rescue experiments revealed that circPDE3B’s role as an oncogene in ESCC depends partly on LAMA1, suggesting that LAMA1 has an essential role as a mediator of circPDE3B/miR-4766-5p axis biological effects in ESCC. Consistent with our findings, LAMA1 is frequently upregulated in multiple cancers, including colorectal carcinoma [27], melanoma [28], gastric cancer [29], and ESCC [30], and plays a crucial role in tumor metastasis. Laminins regulate several cellular activities, e.g., cell migration, adhesion, metastasis, and tumor growth. Laminins are a major component of extracellular matrix (ECM) structures and are involved in tumor cell metastasis [31]. Interestingly, GSVA, GSEA, and KEGG pathway analyses showed that EMT was the most significantly enriched pathway in LAMA1-high expression ESCC tissues. We also observed that both circPDE3B or LAMA1 knockdown suppressed proliferation, invasion, and EMT phenotypes in vitro and in vivo, whereas rescue experiments indicated that co-transfection of miR-4766-5p inhibitor or circPDE3B vector partially attenuated the inhibitory effect, respectively. In summary, these results corroborate our hypothesis that circPDE3B acts as a ceRNA and promotes LAMA1-mediated EMT, metastasis, and proliferation in ESCC by sponging miR-4766-5p.

## Conclusions

Our findings demonstrate that circPDE3B is upregulated in ESCC tissues and that high circPDE3B expression acts as an independent prognostic factor of poor survival in patients with ESCC. CircPDE3B promotes ESCC cell tumorigenesis and metastasis by sponging miR-4766-5p to promote *LAMA1* expression. The circPDE3B–miR-4766-5p–LAMA1 axis regulates ESCC progression by activating EMT. The circPDE3B–miR-4766-5p–LAMA1 axis may therefore act as a biomarker of prognosis and as a therapeutic target in ESCC.

## Methods

### Data collection and processing

We obtained data on RNA sequencing transcriptome and clinicopathological information of 162 patients with ESCC and 11 normal samples from the TCGA database. We obtained circRNAs expression profiles of three pairs of esophageal squamous cancer tissues and the corresponding adjacent non-cancerous tissues from the GEO database (https://www.ncbi.nlm.nih.gov/geo). The GEO accession number is GSE131969. The circRNAs chip (Agilent-069978 Arraystar Human CircRNA microarray V1) for human circRNAs splicing sites was used. Identification of differentially expressed circRNAs and sample cluster analyses were conducted in the R software environment using the DEseq2 and pheatmap R packages (version 3.6.), respectively.

### Human tissue collection

We obtained permission from The First Affiliated Hospital of Zhengzhou University Ethics Committee; all patients provided written informed consent. We collected 92 ESCC tissues and the adjacent normal tissues from the patients during surgery at The First Affiliated Hospital of Zhengzhou University. The patients had received no treatment before the study. We preserved the specimens at −80 °C before use.

### Cell culture

Six ESCC cell lines (ECA109, KYSE-410, KYSE-150, TE-10, TE-1, EC9706) and one normal human esophageal epithelial cell line (Het1A) were purchased from American Type Culture Collection (Manassas, VA, USA). We maintained the cells in Dulbecco’s modified Eagle’s medium (Sigma, St. Louis, MO, USA) that contained 10% fetal bovine serum (Fuheng, Shanghai, China) at 37 °C in a humidified incubator with 5% CO_2_.

### RNA transfection

Three shRNAs against circPDE3B (sh-circPDE3B#1–3), matching NC (sh-NC), shRNA against LAMA1 (sh-LAMA1), and its matching NC (sh-NC) were commercially acquired and synthesized by Readybio (Zhengzhou, China). The miR-4766-5p mimics, miR-4766-5 inhibitor, and their corresponding NCs (inhibitor-NC and miR-NC) were synthesized by Readybio.

### RNA extraction and qRT-PCR

We isolated total RNA from cells or tissues with TRIzol^®^ (Invitrogen, Waltham, MA, USA). We used a Transcriptor First Strand cDNA Synthesis kit (Takara, Beijing, China) according to the manufacturer’s instructions to reverse-transcribe the RNA into cDNA. Thereafter, qRT-PCR was performed to measure gene expression using an SYBR Green kit (Takara). The relative expression level was calculated using the comparative threshold cycle (2^−ΔΔCt^) method, and glyceraldehyde-3-phosphate dehydrogenase (GAPDH) was used as the endogenous reference gene. The primers used in the PCR are listed in Supplementary Table S1.

### Western blotting

Cellular proteins were extracted using radioimmunoprecipitation assay lysis buffer and subjected to sodium dodecyl sulfate-polyacrylamide gel electrophoresis (SDS-PAGE). After transfer, the proteins were blocked with milk and probed with primary antibodies against the following: LAMA1 (Santa Cruz, CA, USA; sc-74418), MMP2 (Santa Cruz Biotechnology; sc-13594), MMP7 (Santa Cruz Biotechnology; sc-515703), MMP9 (Santa Cruz Biotechnology; sc-393859), GAPDH (ABclonal, Woburn, MA, USA; AC002), N-cadherin (Cell Signaling Technology, Danvers, MA, USA; 13116S), E-cadherin (ABclonal; A3044), Snail (Cell Signaling Technology, 3879S), Slug (Abcam, Cambridge, MA, USA; ab51772), and vimentin (Abcam; ab92547) at 4 °C overnight. Then, the membranes were incubated with secondary antibodies and exposed using the LI-COR Odyssey Imaging System (Lincoln, NE, USA).

### Luciferase assay

We seeded HEK293T cells (5000/well) in 96-well plates. After 24 h, the cells were transfected with wild-type and mutant circPDE3B or LAMA1 reporter plasmid and miR-4766-5p mimics. After 48 h, we assessed the relative luciferase activity using a dual-luciferase reporter assay system (Promega, Madison, WI, USA).

### RNA immunoprecipitation (RIP)

The RIP assay used Magna RIP RNA-Binding Protein Immunoprecipitation reagent (Millipore, Burlington, MA, USA). Cells were resuspended in lysis buffer, RNase inhibitor, and protease (Epicenter). The cell lysates were then incubated in AGO2 or control immunoglobulin G (IgG)-coated beads. After incubation with proteinase K, the immunoprecipitated RNA was isolated and measured using qRT-PCR.

### IHC staining

IHC staining was performed as described previously [32].

### Cell proliferation and transwell assays

Cell proliferation ability was examined using the CCK-8, colony formation, and EdU assays, which were performed as described previously [32].

### Tumor formation assay and lung metastasis assay in a nude mouse model

We purchased BALB/c nude mice (male, 6 weeks old) from SJA Laboratory Animal Co., Ltd. (Shanghai, China). The mice received subcutaneous injections of about 5 × 10^6^ cells mixed with 0.2 ml phosphate-buffered saline in their backs. We measured the tumor size and volume regularly. Thirty days later, the mice were sacrificed and the tumors were excised. The tumor mass and volume were measured, and then the tumors were photographed. For the in vivo tumor metastasis assay, we injected cells (1 × 10^6^) into the tail vein of the mice (*N* = 6 per group). Forty-two days later, we determined the lung micrometastases per tissue section of individual mice via morphological observation of hematoxylin–eosin (H&E)-stained sections. The tumor tissues were sectioned, and IHC staining was performed as described previously [32]. The animal experiments followed the Ethical Guidelines for the Use of Laboratory Animals and were approved by the Zhengzhou University Ethics Committee.

### Statistical analysis

The statistical analysis was performed using SPSS 23.0 (SPSS Inc., Chicago, IL, USA). We repeated each experiment at least three times, and report the results as the mean ± standard deviation. Comparisons were made using a two-tailed Student’s *t* test or one-way analysis of variance. We calculated the survival rate using the Kaplan–Meier method.

## Supplementary information

Supplementary Figure Legends

Supplementary Figure S1

Supplementary Figure S2

Supplementary Figure S3

Supplementary Figure S4

Supplementary Figure S5

Supplementary Figure S6

Supplementary table 1

Supplementary table 2

Supplementary table 3

## Data Availability

The datasets used and/or analyzed during the current study are available from the corresponding author on reasonable request.
