# Supplementary material for: Circular RNA hsa_circ_0000277 sequesters miR-4766-5p to upregulate LAMA1 and promote esophageal carcinoma progression
Source: Cell Death Dis. 2021 Jul 5;12(7):676. doi: 10.1038/s41419-021-03911-5 (PMC8257720; doi:10.1038/s41419-021-03911-5)
Supplement: Supplementary file 1 — Supplementary Figure Legends [file 41419_2021_3911_MOESM1_ESM.docx]

Supplementary Figure legends

**Supplementary Figure S1.** The relative expression levels of circular circPDE3B (**A**) or linear PDE3B mRNA (**B**) were analyzed by qRT-PCR after indicated transfection in TE-1 and EC9706 cells. * p < 0.05, **p < 0.01, ***p < 0.001.

**Supplementary Fig. S2.** Expression levels of MMP-2, MMP-7 and MMP-9 in TE-1 and EC9706 cells after indicated transfection were analyzed by western blot.

**Supplementary Figure S3.** Fluorescence in situ hybridization (FISH) were performed to determine the subcellular localization of circPDE3B in ESCC cells. Scale bar: 200 μm.

**Supplementary Fig. S4.** Expression levels of ELOVL2 and ONECUT2 in TE-1 and EC9706 cells after indicated transfection were analyzed by western blot.

**Supplementary Fig. S5.** Representative images and quantification of immunostaining of E-cadherin and N-cadherin in subcutaneous tumor models.(scale bar: 200 μm). * p < 0.05, **p < 0.01, ***p < 0.001.

**Supplementary Figure S6.** Uncropped western blot raw data
