# Supplementary figures and images for: Circular RNA hsa_circ_0000277 sequesters miR-4766-5p to upregulate LAMA1 and promote esophageal carcinoma progression
Source: Cell Death Dis. 2021 Jul 5;12(7):676. doi: 10.1038/s41419-021-03911-5 (PMC8257720; doi:10.1038/s41419-021-03911-5)

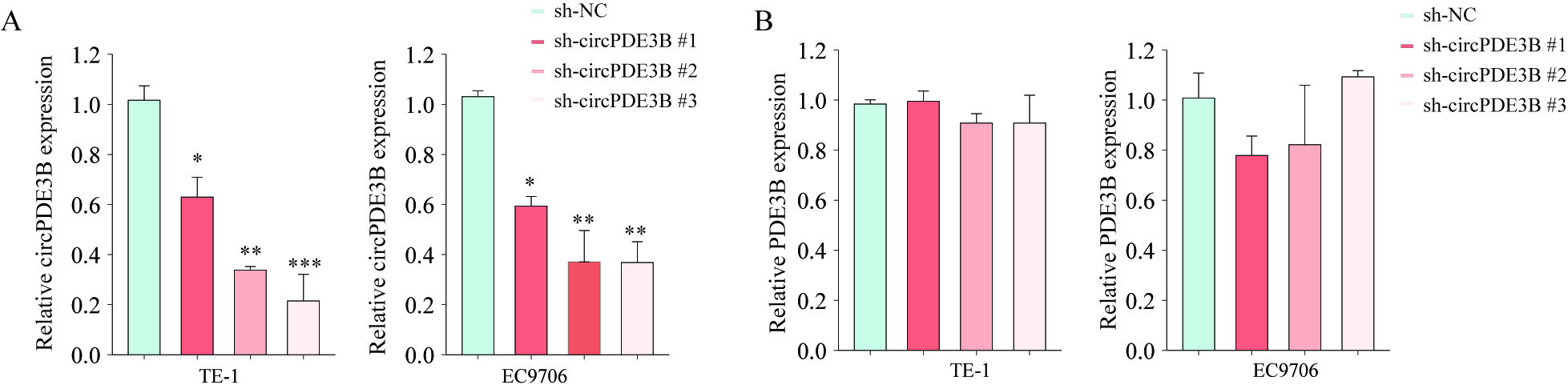

Supplement: Supplementary file 2 — Supplementary Figure S1 [file 41419_2021_3911_MOESM2_ESM.tif]

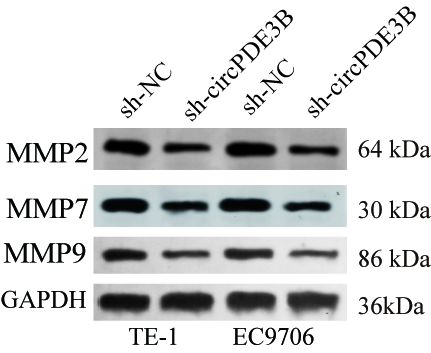

Supplement: Supplementary file 3 — Supplementary Figure S2 [file 41419_2021_3911_MOESM3_ESM.tif]

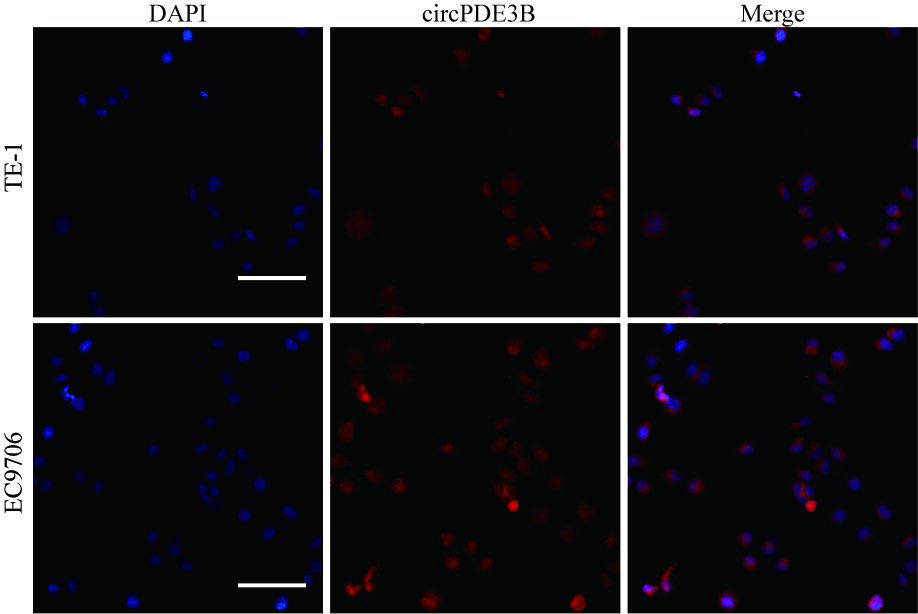

Supplement: Supplementary file 4 — Supplementary Figure S3 [file 41419_2021_3911_MOESM4_ESM.tif]

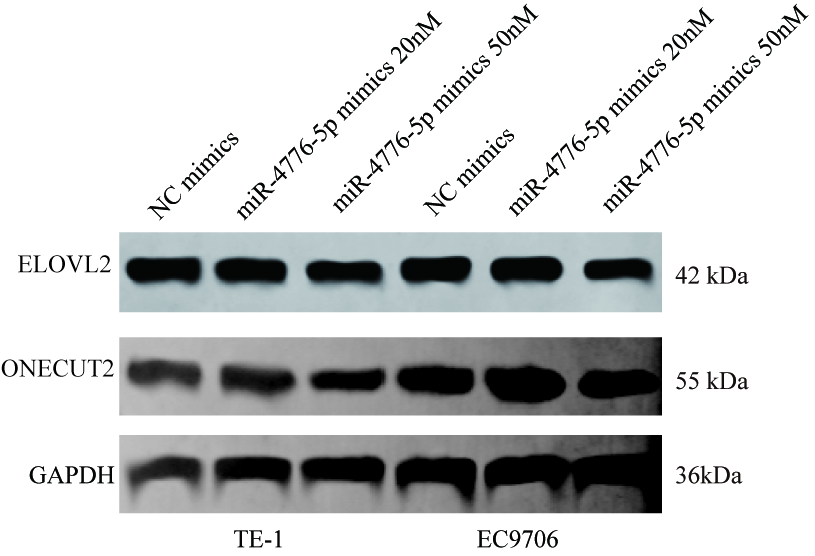

Supplement: Supplementary file 5 — Supplementary Figure S4 [file 41419_2021_3911_MOESM5_ESM.tif]

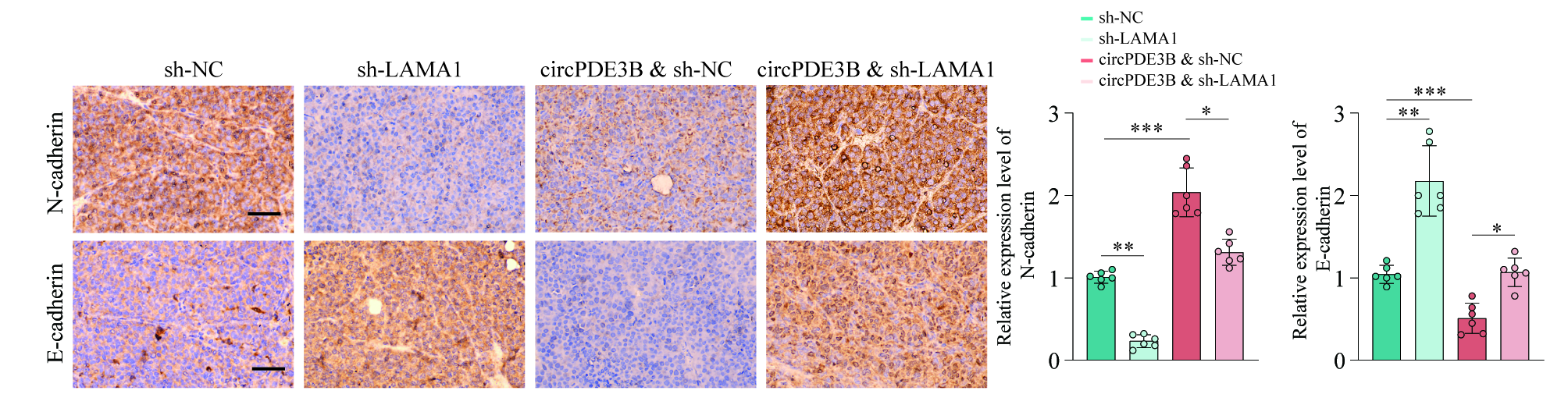

Supplement: Supplementary file 6 — Supplementary Figure S5 [file 41419_2021_3911_MOESM6_ESM.tif]

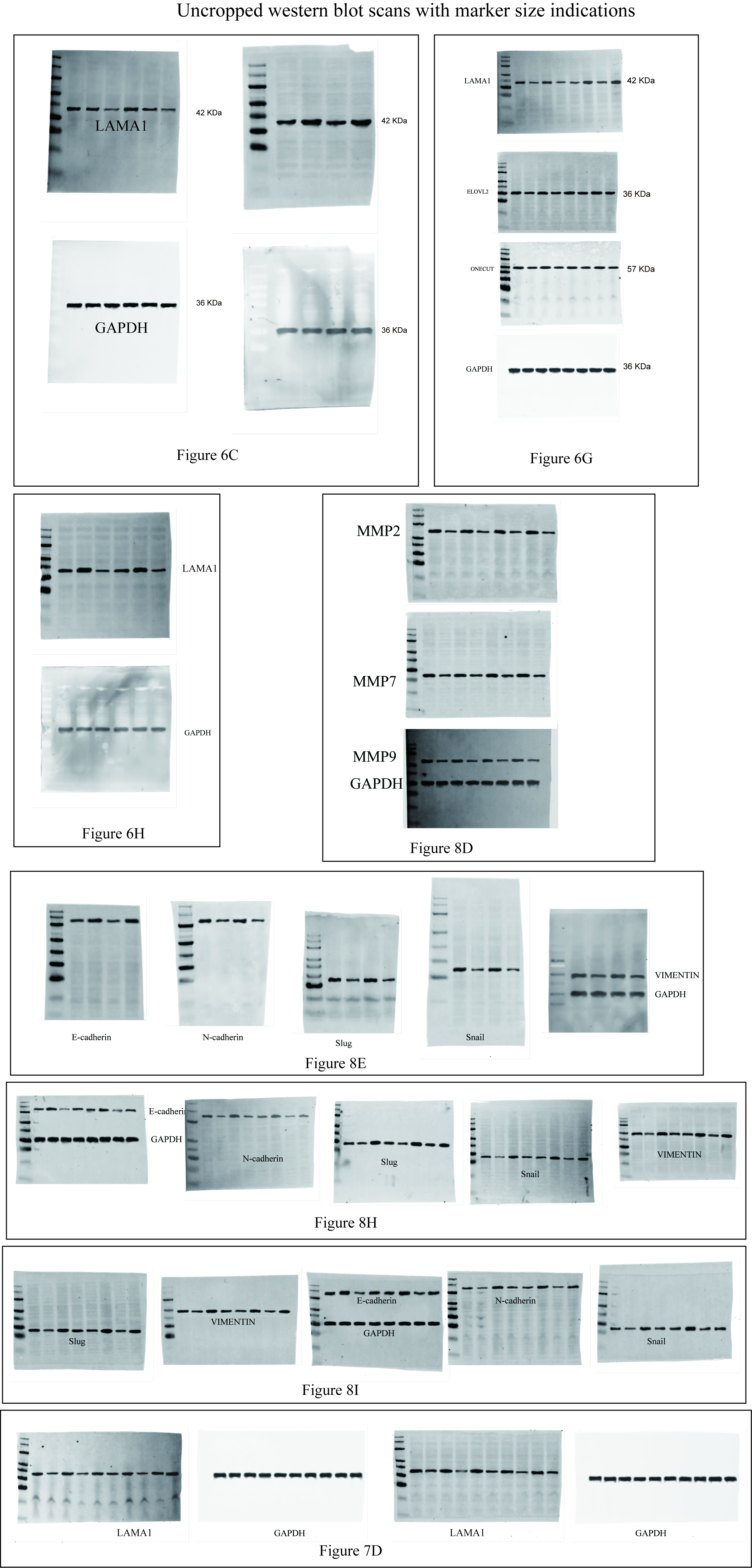

Supplement: Supplementary file 7 — Supplementary Figure S6 [file 41419_2021_3911_MOESM7_ESM.tif]
