# Supplementary material for: Circular RNA hsa_circ_0000277 sequesters miR-4766-5p to upregulate LAMA1 and promote esophageal carcinoma progression
Source: Cell Death Dis. 2021 Jul 5;12(7):676. doi: 10.1038/s41419-021-03911-5 (PMC8257720; doi:10.1038/s41419-021-03911-5)
Supplement: Supplementary file 8 — Supplementary table 1 [file 41419_2021_3911_MOESM8_ESM.docx]

**Supplementary Table S1. Primer sequence**

| Name | Direction | Primer (5'-3') |
| --- | --- | --- |
| hsa_circ_0000277 | Forward | 5'- CAGTCTTCAAGGTGGGATCG -3' |
|  | Reverse | 5'- CTCCATTTCCACCTCCAGAA -3' |
| miR-4776-5p | Forward | 5'-ACACTCCAGCTGGGTTGTGGTTGACGAGA-3' |
|  | Reverse | 5'-CTCAACTGGTGTCGTGGAGTCGGCAATTCAGTTGAG AGACTTTC-3' |
| LAMA1 | Forward | 5′- GTCAGCGACTCAGAGTGTTTG -3′ |
|  | Reverse | 5′- CTTGGGTGAAAGATCGTCAGC -3′ |
| GAPDH | Forward | 5'- CTGGGCTACACTGAGCACC -3' |
|  | Reverse | 5'-AAGTGGTCGTTGAGGGCAATG-3' |
| U6 | Forward | 5′- CTCGCTTCGGCAGCACA-3′ |
|  | Reverse | 5′- AACGCTTCACGAATTTGCGT-3′ |
