# Supplementary material for: Circular RNA hsa_circ_0000277 sequesters miR-4766-5p to upregulate LAMA1 and promote esophageal carcinoma progression
Source: Cell Death Dis. 2021 Jul 5;12(7):676. doi: 10.1038/s41419-021-03911-5 (PMC8257720; doi:10.1038/s41419-021-03911-5)
Supplement: Supplementary file 10 — Supplementary table 3 [file 41419_2021_3911_MOESM10_ESM.docx]

**Table S3. Correlation of clinico-pathological features with circPDE3B expression in ESCC cohort**

|  | Univariate analysis | | |  | Multivariate analysis | | |
| --- | --- | --- | --- | --- | --- | --- | --- |
|  | HR | 95% CI | *P* value |  | HR | 95% CI | *P* value |
| Univariate and multivariate analysis of disease-free survival (n=92) | | | | | | | |
| Age (>60 *vs* <60) | 1.230 | 0.890–1.456 | 0.861 |  |  |  |  |
| Sex (Male *vs* Female) | 0.964 | 0.706-1.461 | 0.964 |  |  |  |  |
| Smoking history (Ever *vs* Never) | 1.267 | 0.881-1.578 | 0.141 |  |  |  |  |
| Lymh node metastasis (Yes *vs* No) | 1.752 | 1.312-2.271 | 0.035 |  | 1.254 | 1.098-1.501 | 0.076 |
| TNM stage (Advanced *vs* Early) | 3.260 | 2.65-4.365 | 0.001 |  | 2.958 | 2.322-3.376 | 0.001 |
| Tumor size (>5cm *vs* <5cm ) | 2.324 | 1.821-2.988 | 0.036 |  | 1.821 | 1.365-2.401 | 0.041 |
| pathology grade (Good *vs* Poor ) | 2.196 | 1.668-2.754 | 0.045 |  | 1.819 | 1.591-2.323 | 0.025 |
| circPDE3B expression (High *vs* Low ) | 2.409 | 1.987-2.985 | 0.175 |  | 2.114 | 1.704-2.413 | 0.035 |
